# A case of *Gemella morbillorum* native valve endocarditis and results of *in vitro* susceptibility testing

**DOI:** 10.1016/j.idcr.2021.e01045

**Published:** 2021-01-09

**Authors:** Farah Tanveer, Joan Pawlak, Dima Youssef, Louis D. Saravolatz

**Affiliations:** Division of Infectious Disease, Department of Internal Medicine, Ascension St John Hospital, Grosse Pointe Woods, MI, USA

**Keywords:** *Gemella morbillorum*, Endocarditis, Antimicrobial susceptibility test

## Abstract

We present a case of a 48 years old male with *Gemella morbillorum* native mitral valve endocarditis. Due to poor growth of the organism, antimicrobial susceptibility test (AST) could not be performed using the CLSI approved method. AST was determined using Etest© strips and the patient was successfully treated with mitral valve replacement and intravenous ceftriaxone.

## Introduction

Infective endocarditis (IE) is an uncommon but life threatening infectious disease with serious complications. Globally, in 2010, IE was associated with 1.58 million disability-adjusted life-years or years of healthy life lost because of death and nonfatal illness or impairment [1]. IE has been classified as “acute” or “subacute–chronic” based on the onset and severity of the clinical presentation and the progression of the untreated disease. The bacteriology of IE varies depending on the cohort examined; however, *Staphylococcus aureus* and Streptococci comprise most of the organisms involved. In recent years, changes in epidemiological profile of infective endocarditis has been noted with emerging species that are often difficult to grow. Infective Endocarditis caused by *Gemella* species remains rare and the literature has been based mainly on case reports. *Gemella* species are gram- positive, catalase- negative, facultative anaerobic cocci which are part of human oropharynx, upper respiratory, genitourinary, and gastrointestinal system flora [2]. These bacteria are easily decolorized during the Gram-staining process, resulting in highly variable gram stain characteristics. The organism's cell wall chemical composition of peptidoglycan is consistent with Gram-positive organisms [3].

## Case

A 48 years old, previously healthy male presented to the outpatient Infectious Disease clinic with an unintentional fifty pounds weight loss, extreme fatigue, drenching night sweats, and progressively worsening shortness of breath with exertion for four months. There was no history of fever, cough, drug abuse, unusual exposures or dental procedures. Physical examination revealed normal vitals but a grade 4/6 systolic murmur in the mitral area. Rest of the physical examination was unremarkable. Laboratory investigations done by his primary care doctor a month prior to this presentation showed normocytic normochromic anemia, and an elevated erythrocyte sedimentation rate at 46 mm/ hr. Complete metabolic panel, thyroid function test, HIV screen and QuantiFERON were normal. Recent computed tomography of chest, abdomen, and pelvis without contrast showed moderate splenomegaly and few areas of diminished attenuation in the spleen representing old infarcts. Blood cultures were obtained and within 48 h grew gram-positive cocci, identified as *Gemella morbilliform*. Patient was admitted and started on intravenous vancomycin and ceftriaxone. Repeat blood cultures 48 h after initiation of antibiotics were negative. Echocardiogram revealed two mitral valve vegetations, 23 × 23 mm and 13 × 8 mm in size, with severe mitral regurgitation requiring mitral valve replacement. Patient was also found to have acute kidney injury secondary to endocarditis associated immune complex mediated glomerulonephritis. To further investigate the source of bacteremia, he underwent colonoscopy which was normal.

The isolate was submitted to a reference laboratory for antimicrobial susceptibility test (AST) but was unable to be performed using CLSI approved method due to poor growth of the organism. We also tried to perform AST in our research laboratory using broth microdilution testing but was unsuccessful as the organism did not grow in the wells when using Mueller Hinton Broth with 5% lysed horse blood [4]. We were able to determine AST results for the *Gemella* sample using Etest© strips. The E-test Application Guide [5] procedure for “Fastidious Gram-Positive Organisms” was utilized. The inoculum was prepared from a 24 h growth and made equivalent to a 1.0 McFarland standard in Brain Heart Infusion Broth. The plates were incubated at 35℃ in 5% CO2.The media recommended by E-test and CLSI was Mueller Hinton agar with 5% blood, but when using this media, we were unable to get sufficient growth to read the results. We were able to get sufficient growth using Tryptone Soya agar with 5% sheep blood, but the organism grew the best on Brucella agar with 5% sheep blood, hemin and vitamin K [6]. With the Brucella agar the growth was confluent and allowed us to easily read the eclipse for the E-test. The E-test application guide recommends Brucella agar for anaerobes. The organism required 48 h of growth to correctly read the results. SPNE ATCC 49,619 was used for quality control as recommended by CLSI. The results are shown in the table.

Intravenous vancomycin was discontinued after the results of the AST, and treatment was continued with intravenous ceftriaxone alone. Patient finished 6 weeks of intravenous ceftriaxone post mitral valve replacement. Renal function gradually improved, and creatinine returned to baseline by the end of treatment. Repeat blood cultures done two weeks post antibiotic therapy were negative. On follow-up visits patient reported significant improvement in his weight, fatigue, night sweats and activity level.

## Discussion

The four species of Gemella that can cause human infections are G. moribillorum, G. haemolysans, G. bergeriae, and G. sanguinis. These species have been implicated in meningitis, brain abscess, endophthalmitis, empyema, blood stream infections, and endocarditis. Gemella morbillorum was initially thought to be part of the genus Streptococcus until 1988 when it was found to be related to G. haemolysans at the genus lever. A recent review of literature suggested that *G. morbillorum* is the most common species associated with endocarditis and mitral valve is the most common heart valve involved. Onset of illness was subacute in most cases and fever was the most common presenting complaint [7]. Predisposing factors for endocarditis include dental infections and procedures, colon malignancies or gastrointestinal diagnostic procedures, immunosuppression, and preexisting cardiac abnormalities [8]. In our patient we were not able to identify any predisposing conditions.

According to published data, the majority of the*Gemella* isolates from various clinical samples are reported to be susceptible to beta lactams and vancomycin. Other antibiotics which can have variable activity include clindamycin, macrolides, levofloxacin, linezolid and aminoglycosides [9,10]. Inherent resistance to TMP-SMX and low-level resistance to aminoglycosides has been reported [9]. Aminoglycosides have been used in combination with beta lactams especially aqueous crystalline penicillin G and ampicillin for synergy and currently this combination is considered the treatment of choice [3,7,11]. Intravenous vancomycin can be used in patients with beta lactam allergy. We did not use aminoglycoside in our patient due to elevated creatinine and he was successfully treated with intravenous ceftriaxone. The susceptibility method reported in this study may assist other laboratories seeking to perform antimicrobial susceptibility test (Table 1).

**Table 1:** *Gemella morbillorum* E -rest results-35 C with CO2.

	Brucella HK	TSA w/ 5% BLD		Brucella HK	Acceptable ATCC
	Agar	Agar	Gemella spp.	Agar	Range
	48 h	48 h	Breakpoint	24 h	
	Final	Final	Susceptible	ATCC 49,619	ATCC 49,619
**Gentamicin**	96	128	NA	32	Not Available
**Vancomycin**	0.38	0.25	≤ 1.0	0.38	0.12–0.50
**Ceftriaxone**	≤ 0.002	≤ 0.002	≤ 1.0	0.032	0.03–0.12
**Ampicillin**	≤ 0.016	≤ 0.016	≤ 0.12	0.064	0.06–0.25
**Clindamycin**	0.094	0.094	≤ 0.25	0.125	0.03–0.12
**Levofloxacin**	0.38	1	≤ 2.0	0.5	0.5–2.0
**Meropenem**	≤ 0.002	≤ 0.002	≤ 0.5	0.064	0.03–0.25
**Doxycycline**	0.032	0.094	NA	0.125	0.016–0.12

## Funding

No funding received.

## Ethical approval

Obtained.

## Consent

Obtained from the patient.

## Author contribution

Analysis and interpretation of laboratory data: Joan Pawlak, Louis Saravolatz

Drafting of manuscript: Farah Tanveer, Dima Youssef

Critical review: Louis Saravolatz

## Declaration of Competing Interest

The authors report no declarations of interest.
